# Deep Learning Model for Accurate Automatic Determination of Phakic Status in Pediatric and Adult Ultrasound Biomicroscopy Images

**DOI:** 10.1167/tvst.9.2.63

**Published:** 2020-12-23

**Authors:** Christopher Le, Mariana Baroni, Alfred Vinnett, Moran R. Levin, Camilo Martinez, Mohamad Jaafar, William P. Madigan, Janet L. Alexander

**Affiliations:** 1 Department of Ophthalmology and Visual Sciences, University of Maryland School of Medicine, Baltimore, MD, USA; 2 Department of Ophthalmology, Children's National Medical System, Washington, DC, USA

**Keywords:** machine learning, ultrasound biomicroscopy, transfer learning, lens status, deep learning

## Abstract

**Purpose:**

Ultrasound biomicroscopy (UBM) is a noninvasive method for assessing anterior segment anatomy. Previous studies were prone to intergrader variability, lacked assessment of the lens-iris diaphragm, and excluded pediatric subjects. Lens status classification is an objective task applicable in pediatric and adult populations. We developed and validated a neural network to classify lens status from UBM images.

**Methods:**

Two hundred eighty-five UBM images were collected in the Pediatric Anterior Segment Imaging Innovation Study (PASIIS) from 80 eyes of 51 pediatric and adult subjects (median age = 4.6 years, range = 3 weeks to 90 years) with lens status phakic, aphakic, or pseudophakic (*n* = 33, 7, and 21 subjects, respectively). Following transfer learning, a pretrained Densenet-121 model was fine-tuned on these images. Metrics were calculated for testing dataset results aggregated from fivefold cross-validation. For each fold, 20% of total subjects were partitioned for testing and the remaining subjects were used for training and validation (80:20 split).

**Results:**

Our neural network trained across 60 epochs achieved recall 96.15%, precision 96.14%, F1-score 96.14%, false positive rate 3.74%, and area under the curve (AUC) 0.992. Feature saliency heatmaps consistently involved the lens. Algorithm performance was compared using 2 image sets, 1 from subjects of all ages, and the second from only subjects under age 10 years, with similar performance under both circumstances.

**Conclusions:**

A neural network trained on a relatively small UBM image set classified lens status with satisfactory recall and precision. Adult and pediatric image sets offered roughly equivalent performance. Future studies will explore automated UBM image classification for complex anterior segment pathology.

**Translational Relevance:**

Deep learning models can evaluate lens status from UBM images in adult and pediatric subjects using a limited image set.

## Introduction

Artificial intelligence-based image recognition models are an expanding field that shows promise to automate the evaluation and diagnosis of ophthalmologic pathologies.^1^ However, these models have focused primarily on fundus photographs, optical coherence tomography (OCT), and visual field analysis,^2^ with relatively little exploration of ultrasound biomicroscopy images.

Ultrasound biomicroscopy (UBM) is an imaging modality that allows for noninvasive, in vivo imaging of structural details of the anterior segment of the eye. This technique is capable of evaluating structures that are normally obscured from direct view, such as the lens-iris diaphragm, or pathologically obscured, as in cases of anterior segment dysgenesis and congenital glaucoma.^3^ Additionally, UBM uniquely offers the ability to evaluate the ciliary body.^4^ Previous case-control studies have shown UBM findings in various anatomic locations, including the ciliary body, iridocorneal angle, and iris, are associated with primary congenital glaucoma.^5^^–^^10^ Although the prognostic and diagnostic impact of these features are still under investigation, there is a current need to produce methods for successful automatic characterization of multiple anatomic locations in UBM to aid these efforts.

A barrier to developing traditional machine learning algorithms de novo for UBM images is the sizeable image dataset often necessary to build a robust model from scratch. Although there are already large image databases available for common diseases, such as diabetic retinopathy^11^ and age-related macular degeneration,^12^ UBM image database development is an ongoing effort, particularly for images of relatively low-incidence congenital and pediatric disease. Transfer learning is a machine learning technique that leverages the weight parameters from a model pretrained on a source task and applies them to a related target task. Transfer learning allows a neural network to train on larger datasets and then fine-tune the output of those models using the smaller medical datasets for the specific target task. Previous studies have demonstrated the ability of convolutional neural networks to identify anterior segment structures on smaller numbers of UBM images with transfer learning.^13^ However, these studies have primarily aimed to assess acute angle closure glaucoma and glaucomatous changes in the adult anterior eye, and have not studied performance in the pediatric population dataset where techniques that improve performance on small datasets would have a significant benefit.

In this study, we seek to demonstrate the feasibility of transfer learning in classifying lens status in pediatric and adult subjects. Automatic lens status classification alone has limited clinical utility; however, previous studies have demonstrated the value of utilizing a simplified task on larger sets of data to generate a model that can be fine-tuned for more specific tasks with necessarily smaller datasets.^14^^,^^15^ A successful lens classification model in UBM images is an important initial step toward models that can recognize and evaluate rare disease states with smaller available datasets, such as pediatric and congenital disease. Additionally, unlike prior studies that are prone to intergrader variability and subjectivity, lens status classification is a ground-truth task that should prompt a model to learn this clinically relevant fundamental task, lens localization, to apply in complex anterior segment pathology classification. For these reasons, lens status classification is an ideal initial task to evaluate the potential of convolutional neural networks in identifying anterior segment anatomic features in UBM images at the depth of the lens and posterior iris.

## Methods

Our cohort included both prospective and retrospective subjects who underwent UBM imaging at participating institutions between December 2014 and December 2019. Prospective subjects were previously consented and enrolled in the multicenter image database, the Pediatric Anterior Segment Imaging and Innovation Study (PASIIS; Baltimore, MD). PASIIS is a collaborative program between the University of Maryland and Children's National Medical Center designed to apply advances in technology and image analysis specifically to clinical evaluation and management of pediatric anterior segment disease. Retrospective subjects were included after review of image database and chart review.

Subject age at the time of examination ranged from 3 weeks to age 89 years (median age of 4.6 years, range = 3 weeks to 90 years) (Table 1). UBM images were obtained using the Aviso Ultrasound Platform A/B UBM with 50 MHz linear transducer (Quantel Medical, Bozeman, MT) or the Accutome UBM Plus Platform with 48 MHz linear transducer (Keeler Accutome, Inc., Malvern, PA). Forty-six of 285 images were collected on the Accutome platform and the remaining 239 of 285 images were collected on the Aviso platform. Lens status composition and resolutions, as well as representative images for each device can be found in Supplementary Tables S1 and S2 and Figures S1 and S2.

Complete UBM image databases of adult images and pediatric images were reviewed from participating institutions for retrospective inclusion. Inclusion criteria included availability of clinical history and central axial UBM images. The only requirement for image quality was the lens (or area where the lens would typically be situated in the case of aphakia) was visible in the frame. UBM was performed by various operators, including ophthalmic photographers, trained technicians, attending physicians, and trainees. Subjects had undergone a variety of types of UBM imaging for various clinical indications (ranging from voluntary participation as a control subject, to clinical evaluation of lens position after cataract surgery, to unrelated evaluation of anterior segment pathology). Most subjects had imaging of both eyes performed. Several adult subjects were enrolled as controls after consent and compensation for time and travel. Images were captured in still image and video clip formats. For video clips, image stacks were exported and reviewed and appropriate images were included.

Most young children and some older subjects were imaged under general anesthesia concurrent with planned surgical procedure. Subjects imaged under general anesthesia were in supine position. The Alfonso eyelid speculum was used for eyelid opening and stabilization. Cotton tip applicators were used to position the globe when needed. Children and adults imaged while awake in an outpatient clinical setting received proparacaine anesthetic drops prior to imaging. Outpatients were imaged in supine or reclined position without eyelid speculum. For these awake subjects, eyelid opening and stabilization was achieved using cotton tipped applicators. Fixation targets and/or verbal instruction was used to position the globe when needed. Prior to imaging, a viscous ocular lubricant gel was applied to the ocular surface. The transducer probe was covered with a water-filled single-use ClearScan probe cover.

Eligible images were de-identified and reviewed by the principal investigator (J.L.A.) and trained clinical research coordinators (M.B. and A.V.). The probe location was determined from the image to be at or near the center of the cornea, and the pupil landmark in view. The direction of the marker and quality of the image were not factors considered for inclusion in this study, provided the pupil could be identified. Lens status was ascertained from chart review of clinical and surgical history.

We cropped the 285 raw images to exclude any text or labeling generated from the native UBM software while maximizing the ocular anatomy in the frame. We then partitioned our dataset into a training dataset that our model would learn from, a validation dataset, which we used to iteratively score our model and prevent overfitting, and a testing dataset that would remain unseen by the model's training process and could be used as an independent evaluation of a model's classification. We used random sampling without replacement to partition subjects, placing 20% of the total subjects in an independent test dataset, 20% of the remaining subjects in a validation dataset, and the subsequently remaining subjects in a training dataset. The overall proportion of pseudophakic, aphakic, and phakic subjects were maintained while partitioning, meaning that for all testing folds there were 1 to 2 aphakic subjects, 7 to 8 phakic subjects, and 4 to 5 pseudophakic subjects. We then balanced our training dataset by randomly oversampling the under-represented classes until there were an equal number of images for each lens status. The entire training set, including the oversampled images, underwent random augmentation that simulated real-world variance, such as horizontal flipping, a modest affine transformation, and contrast and brightness jittering. By selecting these transformations, the model can learn features that are independent of some user variance. These transformations helped mitigate the risk of oversampling leading to overfitting as there was a 6.25 * 10^−6^ chance that the same transformation would be applied to any image. Images in all datasets were uniformly resized to 108 pixels in height by 262 pixels in width and underwent normalization of pixel values in the range of −1 to 1. This final resolution was selected as it represented an approximate balance between the smallest resolution of the images in either height or width in order to prevent up-sampling, while still maintaining an aspect ratio representative of most images. These transformations were randomly re-applied to untransformed images every epoch.

Rather than build a model from scratch that might be prone to overfitting due to a limited sample size, we used a pretrained model, Densenet-121, and then fine-tuned the final layer's parameters and customized a classifier to classify our images. Densenet, a convolutional neural network architecture described by Huang et al.,^16^ has the advantage of efficient accuracy returns for fewer parameters and memory allocation,^17^ making it an ideal starting point for our model. The Densenet-121 model had been pretrained on ImageNet, a benchmark dataset containing over 14 million images with 1000 classes. As the target task of classifying the lens in UBM images differs from the source task of classifying ImageNet color images, we unfroze the further downstream dense block 4's weight parameters while freezing all other earlier layer's parameters. Doing this allowed us to take advantage of the earlier frozen layers to retain general image feature recognition from Densenet and use the deeper layer parameters to classify lens status based on these extracted features.^18^ The final, fully connected linear classification layer used a dropout rate of 0.6 before applying a log SoftMax function to generate the final likelihood of a certain lens class. The final model's unfrozen parameters and classification layer were trained using a stochastic gradient descent optimization at a learning rate of 0.0006 and a momentum of 0.9. We trained the model using a negative log-likelihood loss function for 60 epochs with a batch size of 32. We used early stopping criteria with a patience of 10 epochs to further mitigate overfitting.

**Table 1.** Demographic Data for Each Lens Status Class With Percentage of Subjects for Sex, Age, Race, and Ethnicity

	Phakic	Aphakic	Pseudophakic
Subjects (*n* = 51)	33	7	21
Eyes (*n* = 80)	46	8	26
Sex			
Male	40.63%	71.43%	47.62%
Female	59.38%	28.57%	52.38%
Age			
<10 years old	65.63%	100%	52.38%
10–20 years	9.38%	0%	4.76%
20–30 years	18.75%	0%	14.29%
30–40 years	6.25%	0%	0%
>40 years old	0%	0%	28.57%
Race and ethnicity			
Black	62.50%	100%	57.14%
White	18.75%	0%	28.57%
Hispanic	18.75%	0%	14.29%
Total images (*n* = 285)	177	27	81

**Table 2.** Aggregated Precision, Recall, F1-Scores, False Positive Rate, and Mean AUC for Each Individual Lens Status Class and the Weighted-Average Scores for the All-Ages Analysis

Class	Precision	Recall	F1-Score	False Positive Rate	Mean AUC
Aphakic (*n* = 27)	85.19%	85.19%	85.19%	1.55%	0.991
Phakic (*n* = 177)	96.63%	97.18%	96.90%	5.56%	0.992
Pseudophakic (*n* = 81)	98.75%	97.53%	98.14%	0.49%	0.994
Weighted average	96.15%	96.14%	96.14%	3.74%	0.992

Corresponding ROC curves can be found in Supplementary Figure S4.

**Table 3.** Aggregated Precision, Recall, F1-Scores, False Positive Rate, and Mean AUC for Each Individual Lens Status Class and the Weighted-Average Scores for the Subgroup Modeling for Subjects Under Age 10 Years

Class	Precision	Recall	F1-Score	False Positive Rate	Mean AUC
Aphakic (*n* = 27)	80.77%	77.78%	79.25%	3.25%	0.981
Phakic (*n* = 110)	94.50%	93.64%	94.06%	8.45%	0.984
Pseudophakic (*n* = 44)	93.48%	97.73%	95.56%	2.19%	0.995
Weighted average	92.20%	92.27%	92.22%	6.15%	0.986

Corresponding ROC curves can be found in Supplementary Figures S2 and S3.

**Table 4.** Precision, Recall, F1-Scores, False Positive Rate, and AUC for Models Trained Using Images from Phakic and Pseudophakic Patients Under Age 10 Years Only and Over Age 10 Years Only and Then Tested on Only Under Age 10 Years Patient Images

	Number of Images in				False Positive	
Class	Training/Validation Set	Precision	Recall	F1-Score	Rate	AUC
Under age 10 y only						
Phakic	40 train, 8 val	100.00%	97.37%	98.67%	0.00%	0.995
Pseudophakic	23 train, 6 val	93.75%	100.00%	98.67%	0.00%	0.995
Over age 10 y						
Phakic	54 train, 13 val	97.37%	97.37%	97.37%	6.67%	0.991
Pseudophakic	31 train, 6 val	93.33%	93.33%	93.33%	2.73%	0.992

Corresponding ROC curves can be found in Supplementary Figure S5.

**Figure 1.**
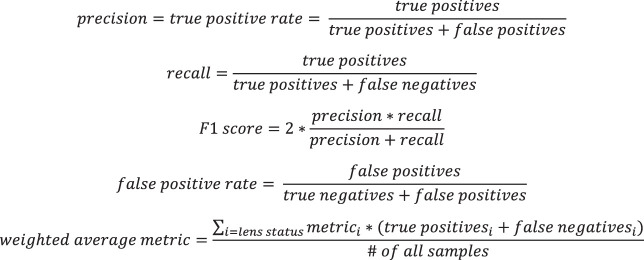
Equations for precision (true positive rate), recall, F1 score, false positive rate, and weighted average metrics.

We performed fivefold cross-validation to evaluate the performance of our model. The original dataset was randomly partitioned into five mutually exclusive testing datasets, and a model was trained on the remaining data for each fold. The composition of images by lens status within each fold is included in Supplementary Figure S3. Each model's predicted labels for testing dataset were aggregated and used to generate a confusion matrix. From these values, we calculated a precision, recall, F1 score, and false positive rate for each lens status using formulas described in Figure 1. Additionally, a receiver operating characteristic curve (ROC) was plotted for each fold and the mean of each fold's true and false positive rate in order to calculate the area under the curve (AUC) and SD. We calculated a weighted-average precision, recall, F1 scores, false positive rate, and AUC to describe the model's overall performance. Finally, we created heat-map visualizations using gradient-weighted class activation mapping to localize regions with high activation. These heatmaps were qualitatively analyzed to assess whether the classification model was activating image regions relevant to lens status evaluation.

**Figure 2.**
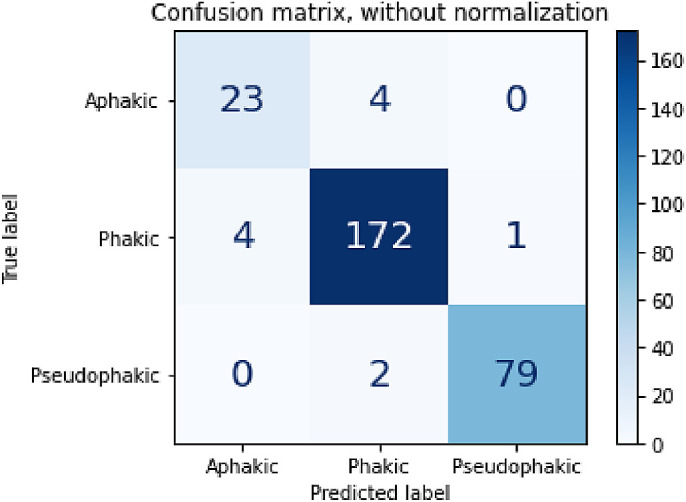
Confusion matrix of the model's classification on all testing set data aggregated across fivefold cross validation. Element (x, y) of this matrix represents the number of UBM images predicted as “x” lens status with a true lens status, “y.”

In order to evaluate whether performance metrics were affected by patient-related factors within the pediatric subset, two experiments were performed. First, a model was trained and evaluated using the subgroup of all patients < 10 years old at the time of the (181 total images; 67 less phakic images and 37 less pseudophakic images and the same number of aphakic images as the all-ages group). The performance metrics and heatmaps of this under 10 subgroup model was compared to the performance of the model that used images from patients of all ages. In the second experiment, two models were trained using 20 subjects (11 phakic and 9 pseudophakic) from 2 conditions: patients < 10 years old at the time of the examination and patients > 10 years old at the time of the examination. These models were then evaluated on a test set of 8 subjects under age 10 (5 phakic and 3 pseudophakic) that had not been included in model training. Aphakic subjects were not included in the modeling, as there were no aphakic subjects over the age of 10 years and the goal was to compare the model's performance when the training set was restricted to patients only above the age of 10 years but tested on images from subjects under 10 years. Other than the parameters involving the number of classes and removing the cross-validation for evaluation, the modeling hyperparameters remained the same as described above.

The source code for the training and evaluation of this model is available at https://github.com/cle801/Lens-Classification.^19^ It is implemented in Python 3.

This study adhered to the ethical principles outlined in the Declaration of Helsinki as amended in 2013. The Institutional Review Board has approved the above referenced protocol. Collection and evaluation of protected health information was compliant with the Health Insurance Portability and Accountability Act of 1996.

## Results

**Figure 3.**
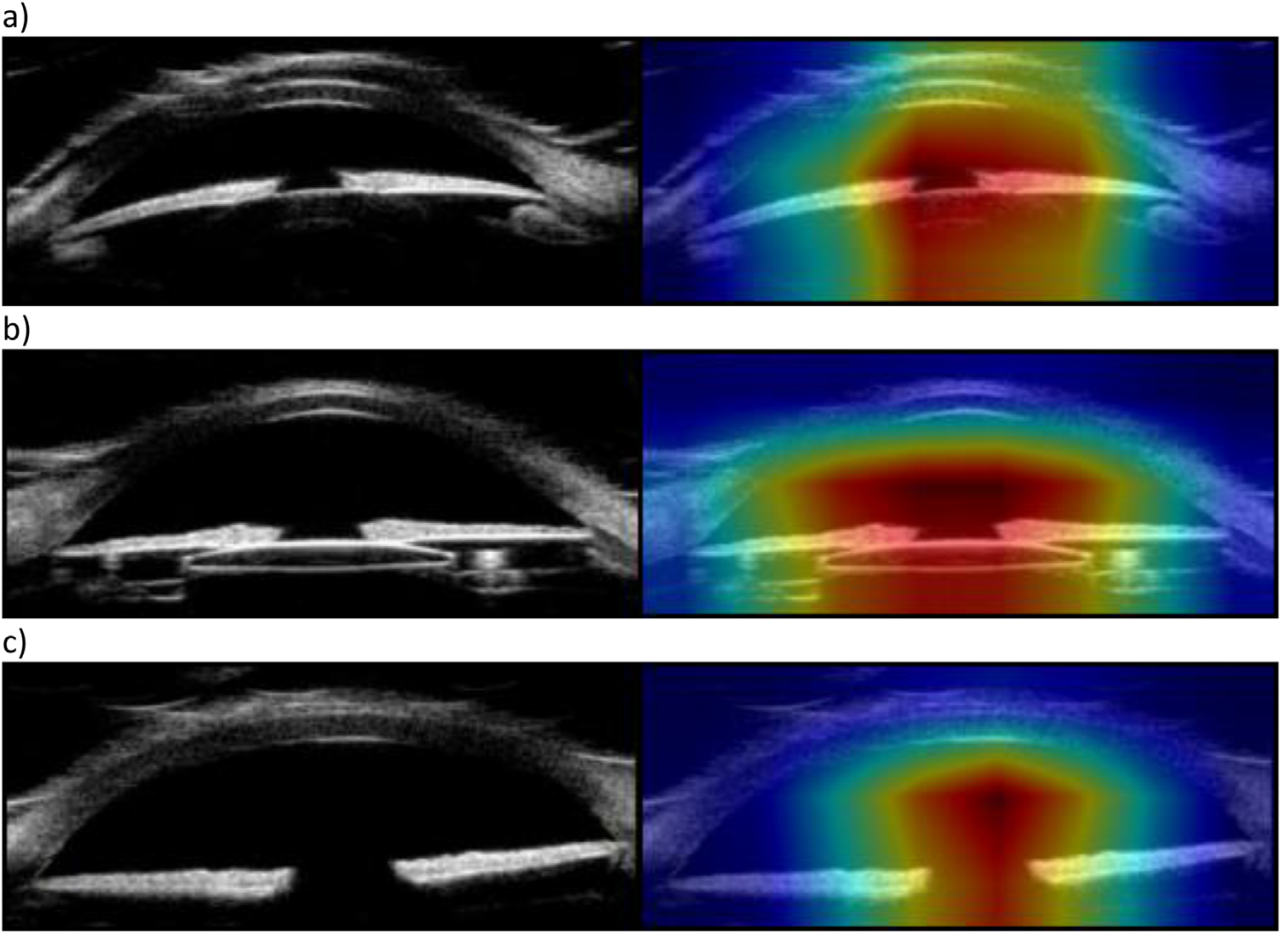
Input images (*left*) and associated feature saliency heat maps (*right*) for correctly identified (**a**) phakic, (**b**) pseudophakic, and (**c**) aphakic images.

Our neural network trained across 60 epochs achieved an aggregated weighted-average recall of 96.15%, a precision of 96.14%, an F1-score of 96.14%, a false positive rate of 3.74%, and an AUC of 0.992 (Tables 2, 3, and 4, Fig. 2). Feature saliency heatmaps generated for testing data consistently involved the lens or adjacent structures (Fig. 3).

**Figure 4.**
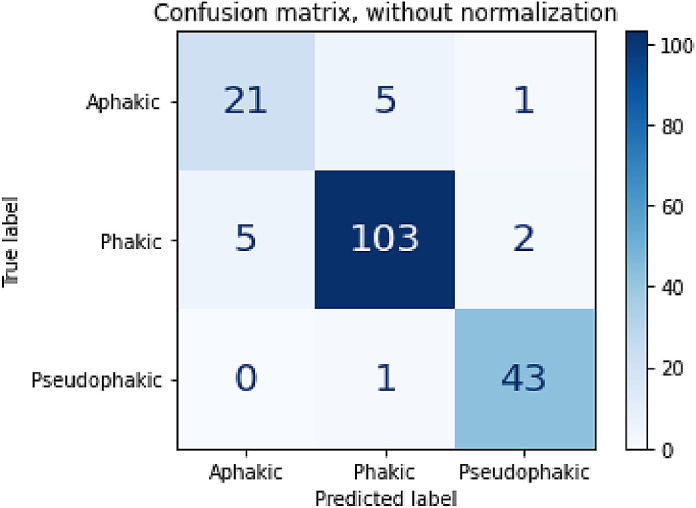
Confusion matrix of the model trained and tested only on patients less than 10 years old.

**Figure 5.**
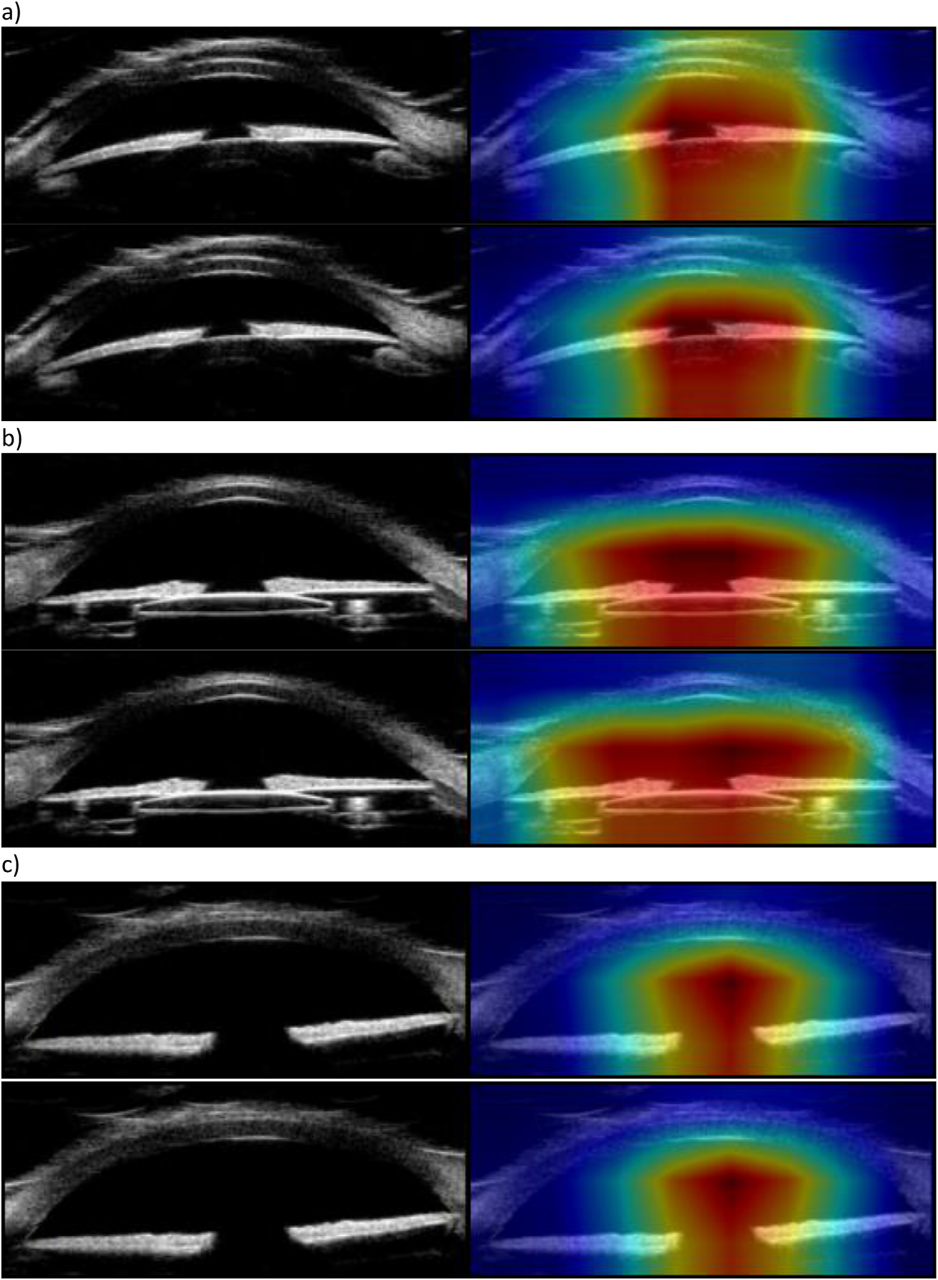
Comparison of the all-age group and the under 10 analysis. (**a**) Phakic comparison demonstrating that the saliency heatmap is more dispersed in the under 10 analysis than the all-ages model. (**b**) Aphakic comparison with a similarly more dispersed heatmap in the under 10 analysis (*bottom*) compared to the all-ages analysis (*top*).

## Discussion

We have successfully demonstrated the feasibility of lens classification in adult and pediatric subjects using convolutional neural network with transfer learning. Our model produced good weighted-average precision, recall, F1-scores, false positive rate, and AUC. The heat maps demonstrate that, for all classes, features most relevant to classification involve the center of the anterior chamber, the pupil, and the central lens.

The rationale for developing this model was as a foundation for future expansion to create algorithms designed to identify complex anterior segment pathology among pediatric subjects with low incidence diseases, such as congenital glaucoma or anterior segment dysgenesis. For pediatric patients with rare diseases, it was necessary to determine the importance of training the algorithm using pediatric versus adult images. An adult image set may be equally effective, and thus extremely useful in training certain features in future models, given the paucity of imaging data from a pediatric population. The under 10-year-old model had slightly worse performance metrics when compared to the all-ages model, except for pseudophakic recall, which had a marginal gain of 0.2%. This is likely due to the smaller size of the training dataset, which was already relatively small. However, in the model using only 181 pediatric images, all weighted-average metrics were still above 90% with an AUC of 0.986 and a weighted-average false positive rate of 6.15%. The model trained only using the under 10-year-old subgroup demonstrated a slight improvement of recall of pseudophakic lens when compared to the all-ages model, likely due to an increased proportion of aphakic images relative to pseudophakic and phakic images within the training dataset. However, the under 10 model had worse overall performance in every other metric, notably a decrease in precision and F1-score, which we attribute to a decrease in overall samples (i.e. 67 fewer phakic images and 37 fewer pseudophakic images) (Fig. 4). Additionally, the comparison of saliency heatmaps in Figure 5 demonstrate, for the same images in Figure 3, the model has slightly larger and thus less lens-specific heatmaps. These results suggest that a larger sample size benefits model performance over a more balanced dataset with fewer oversamples.

**Figure 6.**
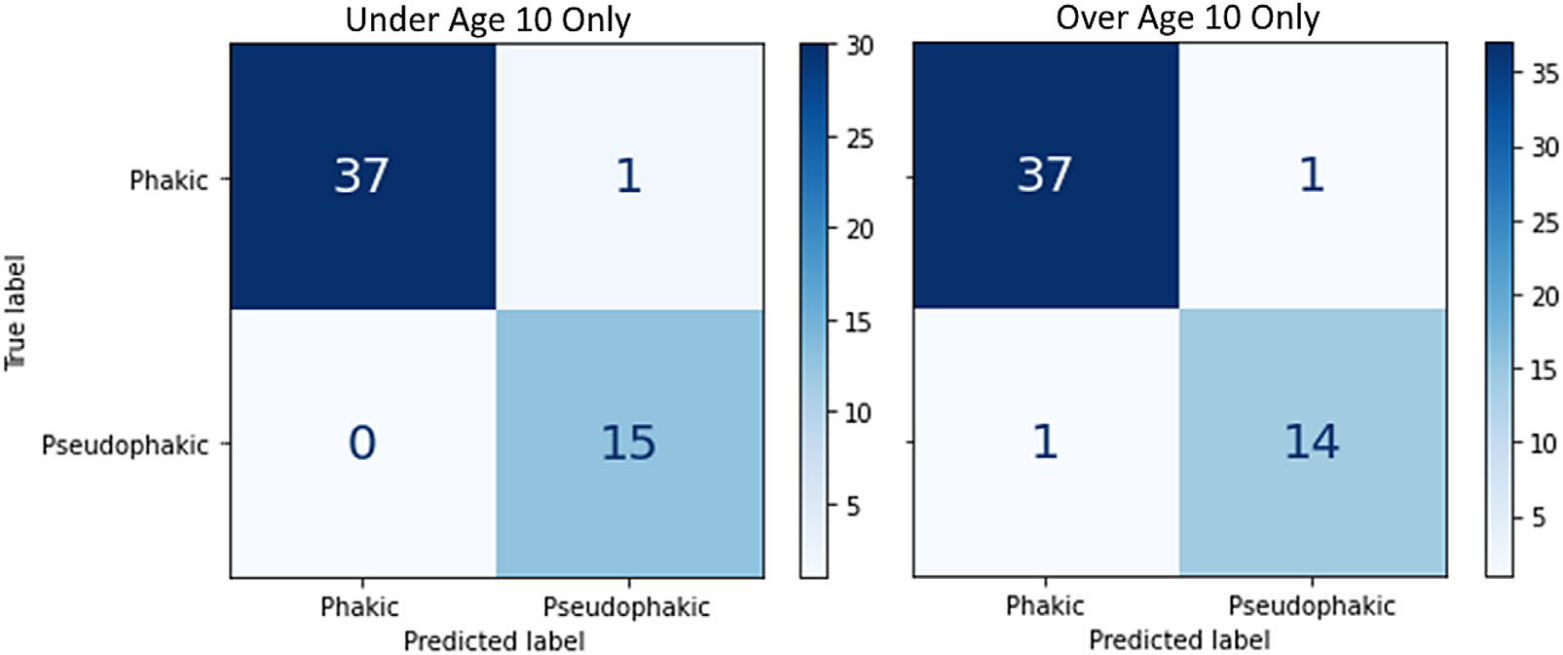
Confusion matrices for models trained using images from phakic and pseudophakic patients under age 10 years only and over age 10 years only and then tested on only under age 10 years patient images.

The models trained on with the same number of patients with pseudophakia and phakia in different age groups performed well on the same testing data set with just under age 10 patient images. There was at most one misclassification per class in both models, although the under age 10 only condition had one less misclassification (Fig. 6). This was consistent with classification errors with all previous models that were largely associated with the aphakic class, which was excluded from this model. These results suggest that the task performance of our modeling method is not dependent on any age-associated features within our data set, at least within the phakic and pseudophakic task.

Training models on labeling tasks that are virtually resistant to mislabeling secondary to grader-dependent diagnostic errors, imperfect gold standard tests, or preclinical disease states, allows for generation of more robust models. As transfer learning continues to develop, we anticipate models, such as this, that classify ground-truth classes within a medical context will be an important intermediate to improve the reliability of further specialized tools within the same medical domain. Ideally, the lens localization features learned by a model trained on a lens status image set could be transferred to future deep learning models trained to identify more nuanced anterior segment pathology from smaller datasets. Additionally, the output of this classification model can be used in an automated combinatory diagnostic model that requires the intentional inclusion of lens status in the decision making. This model's good performance, despite a limited dataset, demonstrates the potential for an approach that automates simple tasks using a small dataset with the hopes of eventually combining the output in a more complex diagnostic workflow.

This study has limitations. The most important limitations were related to features of the image dataset, inclusive of images from various sources, and with an imbalanced number of images among groups. Multiple ultrasonographers contributed to our image database, resulting in image variance in our training and testing image sets. This heterogenous image set is realistic and may have made the algorithm more robust to handle classification of diverse images. On the other hand, use of such variable images for training and testing likely came at a cost to the algorithm's performance. Although all weighted-average metrics were above 95%, the aphakic class precision, recall, and F1-score were lower than the weighted averages. Eight of the 11 total misclassified images were either mislabeled as aphakic images or aphakic images mislabeled as phakic or pseudophakic, suggesting greater difficulty in the model's ability to identify key characteristics of aphakic images. Additionally, our model classified phakia with slightly lower precision than it classified pseudophakia. This model's performance was based on an unbalanced dataset, with relatively few aphakic samples and far more phakic samples. Our image dataset also included only young aphakic subjects. These imbalances are representative of real-world conditions that any lens status classification model would expect to see, as aphakia is uncommon compared to the phakic norm, and primarily seen in pediatric patients. We mitigated the effect of an unbalanced dataset with several strategies, including data augmentation and resampling to expose the model to as many unique aphakic representations as possible. However, it is likely that the trained model's performance was still affected by the imbalance. Although this model's performance is good and thus the number of images is likely adequate, we anticipate that increasing the number of samples would improve the model's ability to classify aphakic images, and therefore its overall performance metrics.

In summary, this algorithm offers a translational step toward generating meaningful UBM image analysis tools that may be clinically useful in pediatric anterior segment disease. The algorithm evaluates at a novel depth of UBM focus (the lens-iris diaphragm, rather than the angle, as has been previously studied using deep learning),^13^ and a novel patient population (pediatric subjects). Future tasks may build upon this first step as a foundation. Although lens status is easily determined without elaborate methods, such as deep learning, this algorithm may provide diagnostic utility when combined with other algorithms in a stepwise format such that lens status informs other options in the differential diagnosis. Through successful automatic localization and classification of lens status in isolation, future automated models that build on a lens status model can explicitly incorporate lens status as a relevant diagnostic factor. For example, the two most common types of pediatric glaucoma are primary congenital glaucoma and glaucoma following congenital cataract surgery. Any algorithm that determined an image that contained features suggestive of pediatric glaucoma would benefit from automated lens status classification to help further differentiate between these subtypes. Therefore, whereas the classification of lens status itself is trivial to most trained humans, lens status plays a role when considering differential diagnoses and therefore has value to be incorporated in future automated models. The same principle can be extended in future studies to other anatomy visualized in UBM beyond lens, such as the ciliary body, and in pathologies beyond pediatric glaucoma in which diagnosis is differentiated along a composite of multiple imaging features. Future work will use lens status classification concurrent with simultaneous evaluation of multiple structures. Lens status can be incorporated into future models as a known clinical factor that can shape pretest probabilities for more complex diagnoses, for example, in distinguishing among common subtypes of pediatric glaucoma. Further studies will be needed to explore the role of automated classification in more complex anterior segment pathology with UBM images.

## Supplementary Material

Supplement 1
