# Supplementary material for: Deep Learning Model for Accurate Automatic Determination of Phakic Status in Pediatric and Adult Ultrasound Biomicroscopy Images
Source: Transl Vis Sci Technol. 2020 Dec 23;9(2):63. doi: 10.1167/tvst.9.2.63 (PMC7779873; doi:10.1167/tvst.9.2.63)
Supplement: Supplement 1 [file tvst-9-2-63_s001.pdf]

Supplemental Materials

Supplementary Table 1: Accutome vs Quantel image composition by lens status and resolution

	Accutome	Quantel
Aphakic	6	21
Phakic	26	151
Pseudophakic	14	67
Min Resolution (pixels)	(101,422)	(241,464)
Max Resolution (pixels)	(630,1039)	(503,1077)

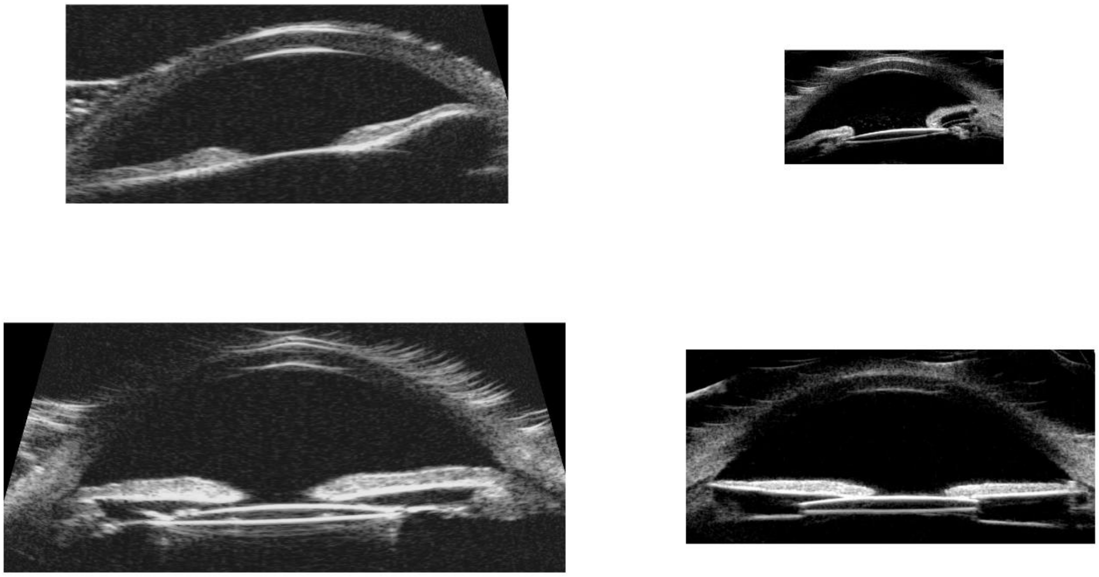

Supplementary Figure 1: Lowest (top) and highest (bottom) resolution cropped images for Accutome (left) and Quantel (right)

Supplementary Table 2: Lens status breakdown and performance metrics for each cross-validation fold for both under-10 and all-ages model

	Fold 1	Fold 2	Fold 3	Fold 4	Fold 5
**Under 10**					
Subjects	10	10	8	7	7
Aphakic Images	14	4	4	3	2
Phakic Images	38	12	17	18	25
Pseudophakic Images	15	8	8	6	7
Total Images	67	24	29	27	34
Precision	93.28%	100.00%	83.18%	96.49%	97.06%
Recall	92.54%	100.00%	79.31%	96.30%	94.12%
F1 Score	92.47%	100.00%	80.15%	95.58%	94.98%
False Positive Rate	5.20%	0.00%	16.31%	7.41%	0.37%
AUC	0.983	0.995	0.964	0.995	0.995
**All Ages**					
Subjects	13	13	12	12	12
Aphakic	14	4	4	3	2
Phakic	21	42	41	27	46
Pseudophakic	11	11	34	11	14
Total Images	46	57	79	41	62
Precision	97.92%	94.06%	97.59%	95.12%	97.31%
Recall	97.83%	92.98%	97.47%	95.12%	96.77%
F1 Score	97.81%	93.25%	97.08%	95.12%	96.91%
False Positive Rate	1.83%	5.60%	2.73%	4.90%	4.69%
AUC	0.995	0.989	0.993	0.994	0.993

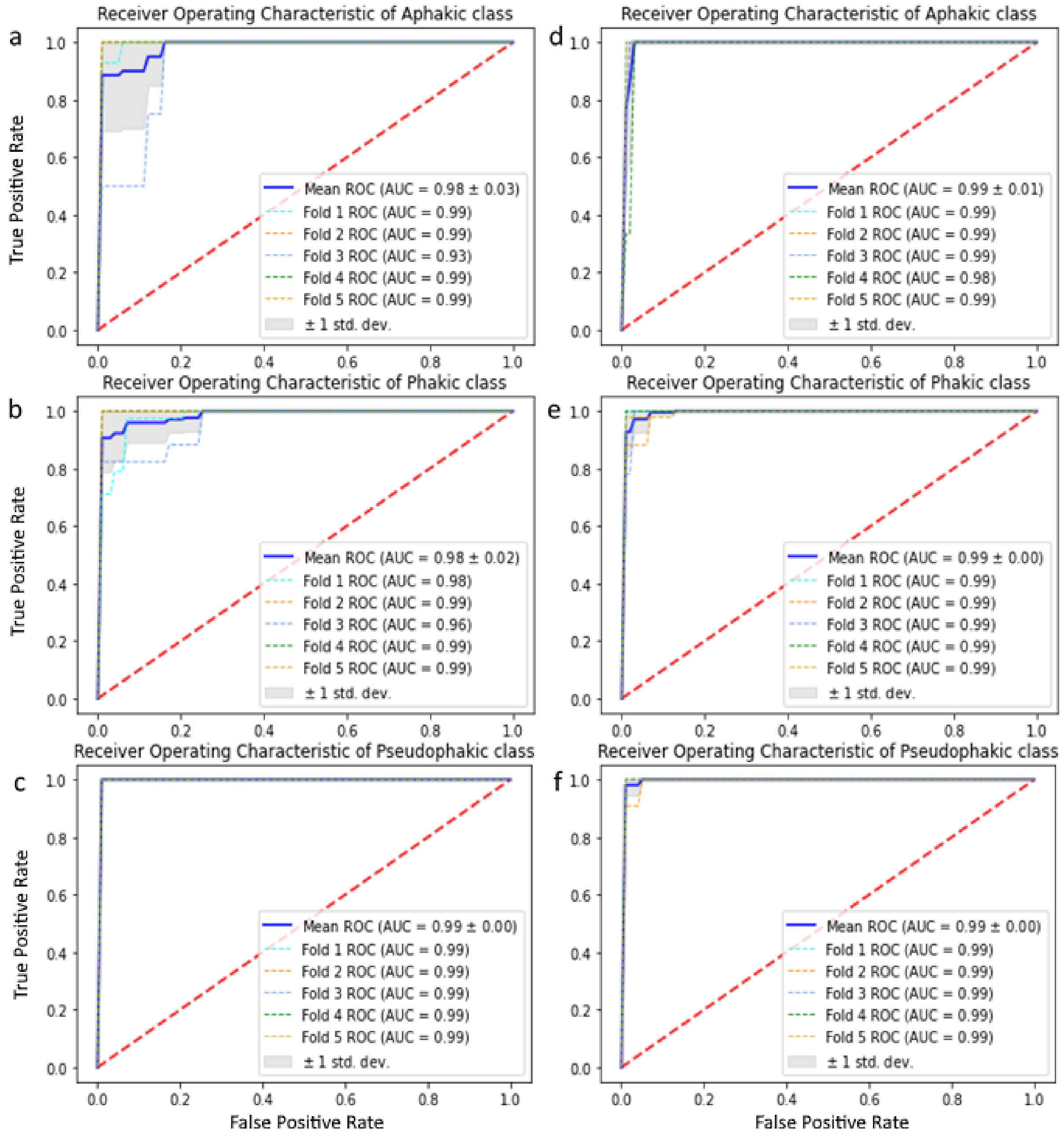

Supplementary Figure 2: ROC curves for each class and fold for both under-10 (left) and all-ages (right) model

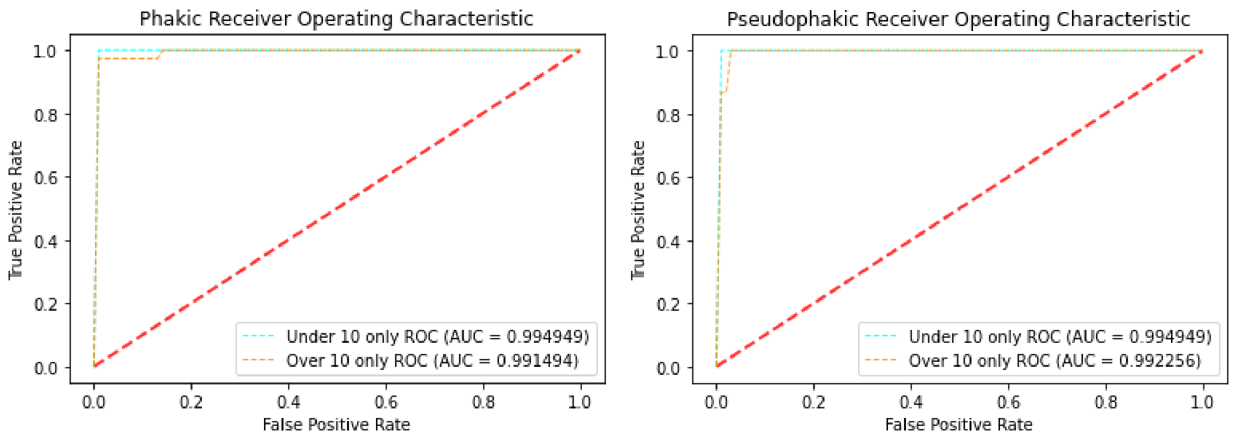

Supplementary Figure 3: ROC curves for phakic and pseudophakic class for under 10 and over 10 only ROC models tested on only under 10 year old patient images.
